# Gastric Emptying Scintigraphy Protocol Optimization Using Machine Learning for the Detection of Delayed Gastric Emptying

**DOI:** 10.3390/diagnostics14121240

**Published:** 2024-06-13

**Authors:** Michalis F. Georgiou, Efrosyni Sfakianaki, Monica N. Diaz-Kanelidis, Baha Moshiree

**Affiliations:** 1 Department of Radiology, University of Miami Miller School of Medicine, Miami, FL 33136, USA; esfakianaki@med.miami.edu; 2 Department of Radiology, Jackson Memorial Hospital, Miami, FL 33136, USA; monica.diazkanelidi@jhsmiami.org; 3 Atrium Health, Wake Forest University, Charlotte, NC 28204, USA

**Keywords:** gastric emptying scintigraphy, machine learning, artificial intelligence, nuclear medicine

## Abstract

Purpose: The purpose of this study is to examine the feasibility of a machine learning (ML) system for optimizing a gastric emptying scintigraphy (GES) protocol for the detection of delayed gastric emptying (GE), which is considered a primary indication for the diagnosis of gastroparesis. Methods: An ML model was developed using the JADBio AutoML artificial intelligence (AI) platform. This model employs the percent GE at various imaging time points following the ingestion of a standardized radiolabeled meal to predict normal versus delayed GE at the conclusion of the 4 h GES study. The model was trained and tested on a cohort of 1002 patients who underwent GES using a 70/30 stratified split ratio for training vs. testing. The ML software automated the generation of optimal predictive models by employing a combination of data preprocessing, appropriate feature selection, and predictive modeling analysis algorithms. Results: The area under the curve (AUC) of the receiver operating characteristic (ROC) curve was employed to evaluate the predictive modeling performance. Several models were developed using different combinations of imaging time points as input features and methodologies to achieve optimal output. By using GE values at time points 0.5 h, 1 h, 1.5 h, 2 h, and 2.5 h as input predictors of the 4 h outcome, the analysis produced an AUC of 90.7% and a balanced accuracy (BA) of 80.0% on the test set. This performance was comparable to the training set results (AUC = 91.5%, BA = 84.7%) within the 95% confidence interval (CI), demonstrating a robust predictive capability. Through feature selection, it was discovered that the 2.5 h GE value alone was statistically significant enough to predict the 4 h outcome independently, with a slightly increased test set performance (AUC = 92.4%, BA = 83.3%), thus emphasizing its dominance as the primary predictor for delayed GE. ROC analysis was also performed for single time imaging points at 1 h and 2 h to assess their independent predictiveness of the 4 h outcome. Furthermore, the ML model was tested for its ability to predict “flipping” cases with normal GE at 1 h and 2 h that became abnormal with delayed GE at 4 h. Conclusions: An AI/ML model was designed and trained for predicting delayed GE using a limited number of imaging time points in a 4 h GES clinical protocol. This study demonstrates the feasibility of employing ML for GES optimization in the detection of delayed GE and potentially shortening the protocol’s time length without compromising diagnostic power.

## 1. Introduction

Gastric emptying scintigraphy (GES) is considered the gold standard in detecting delayed gastric emptying (GE) [1,2], which is essential in the diagnosis of gastroparesis [3]. The widely accepted GES protocol is based on the consensus statement of the Society of Nuclear Medicine (SNM), currently the Society of Nuclear Medicine and Molecular Imaging (SNMMI), and the American Neurogastroenterology and Motility Society (ANMS), published in 2008 [4,5]. These ANMS/SNM[SNMMI] recommendations, aimed to create uniformity in GES methodology, are based on a standardized radiolabeled low-fat egg-white meal of specific caloric content [6] and gamma camera imaging performed at time points 0 h, 1 h, 2 h, and 4 h following the meal ingestion [4]. The normative values for delayed GE were derived by a multicenter trial [7] and defined as gastric retention of >90% at 1 h, >60% at 2 h, and >10% at 4 h [1,6]. An important aspect of this study [7] was extending the imaging up to 4 h, considered critical in detecting delayed GE with higher sensitivity and specificity, a finding that has been well established by multiple studies [8,9,10,11]. The significance of the 4 h imaging is not only in confirming delayed GE using the >10% retention criterion in studies that were exhibiting delayed GE in previous time points, such as the 1 h and 2 h, but also in studies that had normal GE up to 2 h but then “flipped” to abnormal delayed GE at 4 h, as this has clinical implications in terms of treatment options. The method we follow at our institution, referred to as the Miami method (MIA) includes imaging at additional time points of 1.5 h and 2.5 h. Despite the importance of GES in the diagnostic workup for gastroparesis, a main limitation is the prolonged time of repeated imaging up to 4 h, which is inconvenient for the patient and demanding on nuclear medicine resources in terms of equipment and technologist’s time. To this effect, it would be useful to explore approaches for streamlining and optimizing the GES protocol without compromising its diagnostic potential, as it has been established with the 4 h time point in its ability to detect delayed GE. We previously sought to determine if our GES protocol could be optimized and safely terminated earlier than 4 h by retrospectively analyzing data of a large cohort and employing conventional statistical analysis [11]. 

### Artificial Intelligence and Machine Learning in Nuclear Medicine

The recent emergence of AI/ML technologies has been increasingly producing novel applications in the healthcare field, including the nuclear medicine practice, creating new opportunities and bringing about transformative changes [12]. AI/ML impacted the entire nuclear medicine imaging pipeline from acquisition and image generation, reconstructions in SPECT and PET, image processing and analysis including radiomics, quantification, segmentation, and image enhancement (denoising, deblurring, partial volume correction) [13,14,15,16,17,18]. At the basic physics level, AI/ML has generated novel applications related to scatter correction, photon depth of interaction, and time of flight PET imaging [13,16]. Additionally, AI/ML introduced new approaches in computer-aided detection, assisted interpretation, computer-aided diagnosis [13,17,19], as well as for reducing administered dose and radiation exposure to the patient [15]. From the clinical perspective, AI/ML applications span the entire spectrum of nuclear medicine, with the three main areas being: cardiology for myocardial perfusion imaging and detection of coronary artery disease or prediction of major adverse cardiac events [16,20]; neurology for neurodegenerative diseases such as Alzheimer’s, Parkinson’s, and other dementia [16,21,22]; and oncology, where it significantly impacted lesion detection and tracking, radiopharmaceutical therapies with precision dosimetry, personalized treatment planning, and therapy response assessment [12,16,19,23]. AI/ML applications explored new venues in drug discovery, radiopharmacy, and radiochemistry [24], that greatly enhanced the evolving field of theranostics [12,23,25]. Additionally, AI/ML facilitated other benefits of a more administrative and logistical nature in nuclear medicine, such as optimizing resource utilization, patient scheduling, report generation, and other operational improvements and workflow efficiencies [16].

In the present study, we aim to retrospectively analyze GES data by employing machine learning (ML) to generate a model that predicts whether GE will be delayed or normal. To our knowledge, there has not been an AI/ML application in the area of nuclear medicine gastric scintigraphy, and this study can serve as proof of concept for GES protocol analysis and optimization.

## 2. Methods and Materials

### 2.1. Data

The study included a cohort of *n* = 1002 adult patients who underwent GES at our institution based on a standardized Tc-99m radiolabeled low-fat egg-white meal and gamma camera imaging at 0 h, 0.5 h, 1 h, 1.5 h, 2 h, 2.5 h, and, 4 h. GE was calculated using the geometric mean of the anterior and posterior decay-corrected counts obtained from the placement of regions of interest outlining the stomach at the given time points. GE was expressed as percent emptying or retention at the specific imaging time points with respect to the 0 h baseline. The 0.5 h time point is utilized for the evaluation of rapid GE, which is not addressed in this research work. The patient characteristics of our cohort and the distribution of the cases as normal and abnormal are described in Table 1. The Institutional Review Board of the University of Miami approved this study (IRB 20210434), and the requirement to obtain informed consent was waived. All data were handled in compliance with the Health Insurance Portability and Accountability Act (HIPAA) of 1996.

### 2.2. ML Modeling

The JADBio AutoML (version 1.4.132) platform (JADBio, Inc., Crete, Greece) was utilized to develop a model for GES optimization. This software automates the production of optimal predictive models by searching for the best machine learning pipeline, which consists of a combination of data preprocessing, feature selection, and predictive modeling analysis algorithms [26,27]. For preprocessing, the AI/ML system performs standardization to ensure that the input features are scaled to have zero mean and standard deviation of 1. Although the latter does not alleviate the effect of outliers, it is crucial for some predictive modeling algorithms such as SVMs. For feature selection, it tries LASSO regularized regression [28] and the statistical equivalent signatures (SES) algorithm [29]. In general, with feature selection the smallest set of relevant yet non-redundant features are sought. This means that some of them may be filtered out because they were redundant for prediction. Particularly, SES draws inspiration from causal modeling theory and leads to the smallest possible subsets. For modeling, it employs both linear/interpretable algorithms such as ridge regression [30] and decision trees [31] as well as non-linear ones such as random forests [32] and SVMs [33]. The search for the best pipeline is based on grid search and the generalized cross-validation approach, while the BBC-CV algorithm is used to correct the bias in the performance of the final model due to the fact that several pipelines were tried [26].

**Figure 1:**
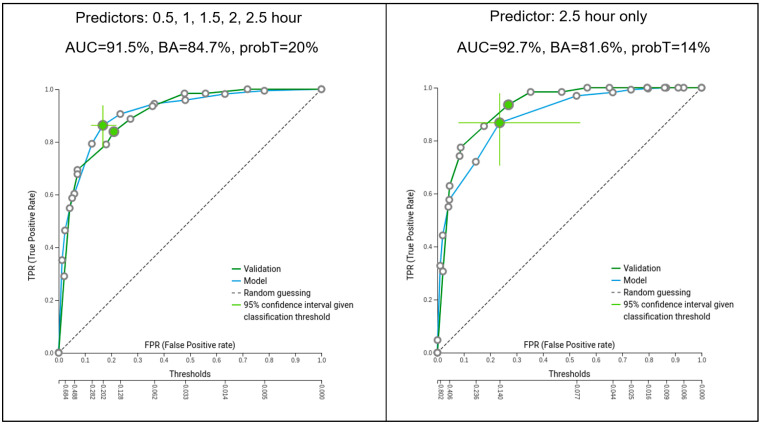
ROC curves of the best performing models optimized for the area under the curve (AUC) using imaging time points 0.5 h, 1 h, 1.5 h, 2 h, 2.5 h (**left**) and single time point 2.5 h only (**right**), as predictors of the 4 h GE outcome. Probability thresholds selected to maximize BA, 20% (**left**) and 14% (**right**) respectively.

**Figure 2:**
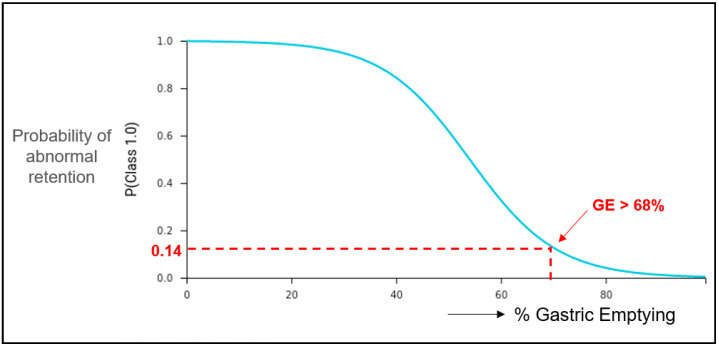
Individual conditional expectation (ICE) plot showing an optimum threshold of 68% gastric emptying at 2.5 h in predicting a normal study at 4 h with probability of 0.86, based on a best-fit model configuration that maximizes balanced accuracy (BA).

**Figure 3:**
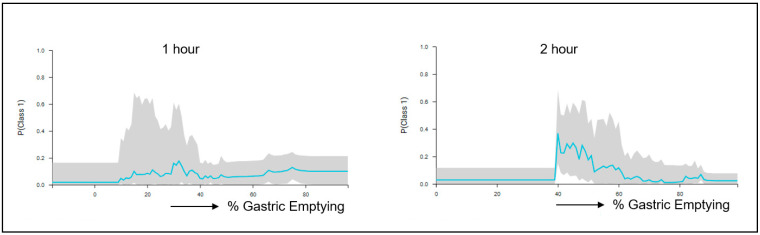
ICE plots of a best performing model in predicting cases that have normal gastric emptying (GE) at 1 h and 2 h, respectively, but then “flip” with delayed GE (>10% retention) at 4 h. At the 1 h time point, (**left**), the model shows the probability increasing moderately in the range of 10% to 40% GE. At the 2 h time point, (**right**), the model shows the probability to markedly increase in the range of 40% to 60% GE.

We used the percent GE at 0.5 h, 1.5 h, 2 h, and 2.5 h as input features and a binary outcome of normal/abnormal using the criterion of >10% retention at 4 h to indicate delayed GE (abnormal). The original class distribution of the entire cohort included 207/1002 cases with abnormal retention (>10% at 4 h) and 795/1002 with normal retention (<10% at 4 h), which was approximately a 20% ratio of delayed GE. Stratified splitting with a ratio of 70/30 was used for the training data set versus the testing one, which is considered an acceptable standard for similar models. The ML system was tested initially using the entire sequence of the imaging time points (0.5 h, 1 h, 1.5 h, 2 h, 2.5 h) as predictors of the 4 h outcome, and, subsequently the 2.5 h, 2 h, and 1 h time points were used by themselves as specific predictors (results shown in Table 2 and Figure 1). Additionally, we tested the predictive accuracy of the ANMS/SNM[SNMMI] standards of gastric retention >90% at 1 h and >60% at 2 h respectively to determine how well they performed using our patient cohort (results shown in Table 3). In the above-described analyses, different types of ML modeling were used to generate the results. During training by AutoML, several ML pipelines consisting of different algorithms and hyperparameter values were tried in a systematic way until the best one was automatically selected. Another testing scenario involved finding the optimal GE threshold at the 2.5 h imaging time point to best predict that the 4 h outcome would be normal with <10% retention (results shown in Figure 2). Furthermore, the ML model was tested with identifying “flipped” cases that had normal GE at 1 h and 2 h but became abnormal with delayed GE at 4 h (results shown in Figure 3).

The area under the curve (AUC) of the receiver operating characteristic (ROC) curve was used in order to find the optimal ML model during the training phase. The optimal operating point of the model along the ROC curve was determined based on balanced accuracy (BA), which is defined as the arithmetic mean of sensitivity and specificity. This metric is commonly used to evaluate the performance of binary classifiers dealing with imbalanced data. The performance of the model was also evaluated in both training and testing using the statistical metrics of sensitivity, specificity, and positive predictive value (PPV) (referred to also as precision). Individual conditional expectation (ICE) plots were utilized to find the optimal thresholds of gastric retention at the various time points and their predictive accuracy for the 4 h outcome.

## 3. Results

Using all the time points as input features, the generated model scored an AUC of 91.5% on the training data and 90.7% on the previously unknown test data. The associated sensitivity, specificity, PPV, and BA were also obtained and tabulated (Table 2). According to feature selection, the 2.5 h time point held sufficient statistical significance to render the earlier time points redundant. When the 2.5 h by itself was used as a predictor of the 4 h GE outcome, the performance was slightly improved compared to using all the time points, with an AUC of 92.7% in training and 92.4% in testing, respectively, pointing to the statistical power of the 2.5 h as a primary predictor of the outcome. The performance of the system by using the 2 h and 1 h individually as predictors of the 4 h outcome was also obtained (Table 2). As demonstrated, the accuracy of the system increases when later imaging time points are used as predictors of the 4 h GE outcome. Supplemental Table S1 provides the description of the best performing model pipeline in terms of preprocessing, feature selection, and predictive algorithm for the analyses corresponding to using all the imaging time points, or the 2.5 h independently, as predictors, respectively. The corresponding confusion matrices are shown in Supplemental Table S3.

Using the thresholds for delayed GE at imaging time points 1 h (retention > 90%) and 2 h (retention > 60%), respectively, which apply to the ANMS/SNM[SNMMI] guidelines, the ML model yielded an AUC of 81.7% for 1 h versus 90.0% for 2 h, and a BA of 62.1% at 1 h versus 78.5% at 2 h. The results for this analysis are shown in Table 3.

We sought also to obtain the GE threshold at 2.5 h that would maximize the BA in predicting the 4 h outcome as delayed with >10% retention. The model determined that a GE of 68% (or 32% retention) at 2.5 h would maximize the BA in predicting normal emptying at 4 h for our patient population, with a probability threshold of 0.86. This is illustrated in Figure 2 using an ICE plot, which is a graphical display that provides visualization of the effect of a specific feature, in this case the GE at 2.5 h, on the prediction of the 4 h outcome as being normal. 

Another GE class that was examined using the ML system involves cases that have normal emptying at 1 h (<90% retention) and 2 h (<60% retention), Tougas et al. [6], respectively, but they then “flip” and present delayed GE at the 4 h marker with >10% gastric retention. Two models were produced for this predictive analysis: a best performing and a best interpretable one; the two models used different best feature selection methods in optimizing their performance, with the best performing utilizing both the 1 h and 2 h GE values, whereas the best interpretable model used only the 2 h GE values. The best performing model indicated that at the 1 h time marker the probability of flipping increases moderately when the GE range is between 10% and 40%, and at the 2 h time marker the probability of flipping increases considerably in the GE range between 40% and 60% (Figure 3). Supplemental Table S2 provides additional information regarding the model pipeline that was utilized by the ML system for the analysis of the 2 h normal GE value in being predictive of “flipping” at the 4 h as abnormal (delayed) GE.

**Figure 4:**
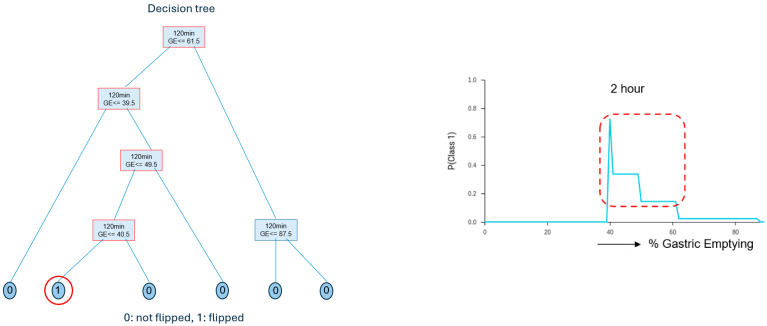
The best interpretable model, which was a decision tree, for predicting at 2 h the probability of “flipping” from normal to delayed GE at 4 h. The decision tree model (**left**) and the plot (**right**) showing that a GE value of 40% at 2 h increases significantly the probability of flipping to a delayed study at 4 h.

The best interpretable model, using GE data only for the 2 h time point, demonstrates that the probability of cases to flip increases in the 40% to 60% GE range (Figure 4). The plateau in the plot after GE of 40% shows the significant increased probability for flipping, even though the flipping information was unknown to the model.

## 4. Discussion

GES based on a 4 h imaging protocol with a standardized radioactively-labeled meal is considered the gold standard in detecting delayed GE, which is a major indication for diagnosing gastroparesis. The prolonged imaging time, which is inconvenient for both the patient and the facility, is a contributing factor in limiting its clinical utility in favor of other methods that may be less effective. As such, several studies were conducted to explore the ways that GES protocols could be optimized by assessing the diagnostic power of several imaging time points individually or collectively, typically using statistical methodologies with a focus on how well the later time points (2 h, 2.5 h, and 3 h) correlate with the 4 h one [11,34,35,36]. To our knowledge, the current work represents the first study where AI and ML were employed in studying the predictive capabilities of the various GE imaging time points with respect to the 4 h outcome. We aimed to harness the potential of AI/ML in learning using multivariate processing without a priori assumptions regarding the distribution of the data and its generalizable capacity in making predictions as accurately as possible, in contrast to traditional statistical methodologies that are based on inferences between the variables used and an assumed modeled distribution [37,38].

In this study we examined the feasibility of an ML system in aiding potential optimization of a 4 h clinical GES protocol. The developed model was constructed using an AI/ML platform (JADBio AutoML). The ML receives as input features the percent GE at imaging time points 0.5 h, 1 h, 1.5 h, 2 h and 2.5 h, and, following training with a large sample of data, it can make predictions with associated statistical power as to whether a GES study would be normal or abnormal, with delayed GE (i.e., >10% retention at 4 h). In one testing scenario, the ML system demonstrated a robust performance with good accuracy, as reported using ROC/AUC, in predicting the status of a GE study whether normal or abnormal (delayed), using all the time points as input features. When the model used the 2.5 h time point by itself as a predictor, the yielded accuracy was slightly improved. This was accomplished using feature selection, which is an integral method of the automated workflows of the ML platform and illustrates the potential superiority of using AI/ML versus conventional statistical methodologies. It also illustrates that the individual time point of 2.5 h has a higher diagnostic power than the linear combination of the earlier time points, a finding that has a causal interpretation.

Additionally, the ML system calculated the predictive power of the nationally accepted GE standards for 1 h (>90% retention) and 2 h (>60% retention) when used as single predictors in detecting delayed GE for the study’s population. The 1 h imaging was maintained in this analysis because it is listed as an imaging time point in the consensus statement [4], even though clinically it does not provide great significance in evaluating delayed GE. The importance of this analysis when performed using the ML platform is that the system can determine, through the application of the automatically selected best-fit model, the optimal applicability of such GE thresholds as predictors of the outcome based on such objective metrics as AUC, sensitivity versus specificity, accuracy, and a corresponding calculated probability. This optimization process can have implications for clinical decision making specific to the study’s specific population, in terms of how confidently a particular case can be considered as potentially becoming delayed at 4 h based on these earlier time points.

The ML system could also determine the optimal GE threshold level for a specific imaging time point as predictor of the 4 h outcome, such as GE of 68% at 2.5 h, with corresponding sensitivity, specificity, and precision, when the selected criterion is maximizing balanced accuracy. This type of analysis applied to later imaging points, as in our protocol of the 2.5 h, can lead to clinical decisions such as earlier termination of the study based on given confidence levels and the associated probability that the study will be delayed or normal. This can result in workflow efficiencies for the facility and convenience for the patient.

The system was also tested for the challenging situation to detect cases that have normal emptying at 1 h and/or 2 h but then “flip” and become abnormal with delayed GE at 4 h. Two models were generated, a best performing and a best interpretable, both with very similar predictive powers but different performance characteristics that could be customized to the best interest and usefulness of the user. This analysis pointed to a particularly increased probability of flipping for a GE value in the range of 40–60% at the 2 h marker, a finding that can be useful in studying the importance of temporal changes in GE and how this can be potentially useful in interpreting clinical symptomatology associated with the patient. Many patients with gastroparesis-like symptoms, or dyspepsia, who may have normal GE are symptomatic at the 2 h scan but not at 4 h, possibly due to impairment in gastric accommodation rather than gastric emptying delay, and, therefore, the change in emptying delay in either direction may be a sign of gastric dysrhythmia (not related to GE) rather than antral hypomotility that gastric delay signifies. Patients with gastric dysrhythmia respond to neurostimulation with gastric electrical stimulators rather than prokinetics. Therefore, we hypothesize that the determination of the GE threshold may have clinical relevance for the prediction of outcomes to various therapies. Such analysis of the inter-relationships between time points in making predictions is an advantage of the AI/ML approach versus as previously applied by our group [11] method using conventional statistics, which could determine the diagnostic power of individual time points as predictors of the 4 h outcome but could not provide flipping information.

Overall, the developed AI/ML system provides the ability to examine GES cases by feeding the model with the percent of GE at various time points for their potential to become delayed at 4 h and the associated accuracy. The concept of employing ML for this analysis is innovative and is introduced with the idea that it can be applied for GES studies that use a 4 h imaging protocol using configurations that are customized to the specific facility and patient population. As an outcome, decisions can be made regarding protocol optimization, such as shortening the total acquisition time interval and thus providing patient convenience and workflow efficiencies for the facility.

The study has some limitations, such as not incorporating the patient’s gender and age, both parameters considered as confounding factors in GES. The ML design is flexible and can be augmented to include these parameters or additional features as inputs. Another limitation is that the study considered the 4 h GE with less than 10% retention as the standard of truth for classifying the cases as normal vs. delayed GE but did not include a clinical correlation for patients’ symptoms or diagnosis. This is because the focus of this study is to demonstrate the feasibility of the AI/ML system in analyzing GES data for its predictive capacity to classify normal vs. abnormal based on a discriminatory metric, in this case the percent retention at 4 h. The model design provides for adaptive training whereby a clinical diagnosis or symptomatology could be additional features to be considered as inputs or outputs.

## 5. Conclusions

This study explored the feasibility of an AI/ML system for detecting delayed gastric emptying in GES. The model was applied to a large patient cohort who underwent GES based on a 4 h imaging protocol and a standardized radioactive meal and could predict the 4 h retention would be greater than 10% by examining the values of GE at earlier imaging time points. The successful application of this AI/ML model can serve to provide potential optimization to the GES protocol by reducing the total acquisition time with resulting patient convenience and savings in resource allocation for the facility performing the study. This in turn can facilitate a wider acceptance for the application of the standardized GES protocol. Furthermore, this novel approach of employing AI/ML serves as a proof of concept for other similar studies with imaging data acquired over several time points that can benefit from protocol optimization.

## Figures and Tables

**Table 1:** Characteristics of the patient population including the number of normal vs. delayed gastric emptying.

No, Gender, Age, Normal/Delayed GE	All	Male	Female
Patients (*n*)	1002	299	703
Mean Age (y)	52	52	52
Median Age (y)	53	54	53
Range (y)	18–91	18–87	18–91
Normal GE < 10% at 4 h	795	249	546
Delayed GE > 10% at 4 h	207	50	157

**Table 2:** Performance of the ML model at various time points as predictors of the 4 h outcome for delayed GE. The column “Estimate” refers to the performance on the training set and the column “Test” refers to the performance on a previously unknown data set. The 95% confidence interval of the training performance is included in brackets.

Predictor for GE	All (0.5, 1, 1.5, 2, 2.5 h)		2.5-h		2-h		1-h	
Metric	Estimate	Test	Estimate	Test	Estimate	Test	Estimate	Test
AUC	91.5% [87.6–95.0%]	90.7%	92.7% [89.2–95.7%]	92.4%	89.5% [84.8–93.4%]	90.0%	78.8% [74.7–82.5%]	81.7%
Sensitivity	86.3% [77.8–93.8%]	83.9%	86.8% [70.6–97.9%]	93.5%	83.5% [68.6–97.2%]	80.6%	68.8% [50–77.8%]	69.0%
Specificity	83.2% [78.1–87.6%]	79.0%	76.4% [46.2–92.1%]	73.1%	76.7% [49.8–87.6%]	82.8%	72.6% [64.4–87.8%]	72.9%
PPV (Precision)	57.9% [48.3–67.9%]	51.0%	55.7% [43.6–73.5%]	47.5%	50.7% [36.9–65.1%]	54.9%	40.2% [33.6–53.7%]	39.8%
Balanced Accuracy	84.7% [79.9–89.4%]	80.0%	81.6% [62–88.7%]	83.3%	80.1% [73–85.2%]	81.7%	70.7% [66.5–74.8%]	70.9%

**Table 3:** Performance of the ML system in predicting the 4 h outcome to be delayed GE using the standard retention thresholds per ANMS/SNM[SNMMI] guidelines at 1 h and 2 h. The column “Estimate” refers to the performance on the training set (95% CI) and column “Test” refers to the performance when tested with the previously unknown data set. The *p* values in parentheses indicate the probability of being abnormal at 4 h given that the gastric retention at the specific time points of 1 h and 2 h is greater than 90% and 60% respectively.

Threshold for Delayed GE	Retention > 90% at 1 h ~P(0.5)		Retention > 60% at 2 h ~P(0.25)	
Metric	Estimate	Test	Estimate	Test
AUC	78.8% [74.7–82.5%]	78.6%	89.5% [84.8–93.4%]	89.6%
Sensitivity	26.7% [13–35.5%]	29%	71.2% [51–86.9%]	59.3%
Specificity	96.6% [94.9–98.7%]	96.6%	86.4% [79.3–94.8%]	94.1%
PPV (Precision)	67.7% [53.3–83.7%]	66.7%	60.8% [47.5–78%]	72.3%
Balanced Accuracy	61.6% [55.6–66%]	62.6%	78.8% [69–85.2%]	86.9%

## Data Availability

The data that support the findings are contained within the article and Supplemental Materials. Further inquiries should be directed to the corresponding author.

## Supplementary Materials

The following supporting information can be downloaded at: https://www.mdpi.com/article/10.3390/diagnostics14121240/s1, Table S1: Description of the best performing ML model pipeline for the various predictive analyses corresponding to the specific features of gastric emptying values at the various imaging time points throughout the GES study. Table S2: Description of the best performing ML model pipeline using the 2 h gastric emptying value as a predictor for “flipping” from normal emptying at 2 h to abnormal (delayed) at 4 h. Table S3: Confusion matrices for the best performing models with the operating points set to maximize balanced accuracy, using all imaging time points as predictors (top) and using the 2.5 h only (bottom).
