# Supplementary material for: Gastric Emptying Scintigraphy Protocol Optimization Using Machine Learning for the Detection of Delayed Gastric Emptying
Source: Diagnostics (Basel). 2024 Jun 13;14(12):1240. doi: 10.3390/diagnostics14121240 (PMC11202747; doi:10.3390/diagnostics14121240)
Supplement: Supplementary file 1 [file diagnostics-14-01240-s001.zip › diagnostics-2906931-supplementary.pdf]

diagnostics MDPI

Supplemental Table S1. Description of the best performing ML model pipeline for the various predictive analyses corresponding to the specific features of gastric emptying values at the various imaging time points throughout the GES study.

Input Feature(s)	Model Pipeline		
	**Preprocessing**	**Feature Selection**	**Predictive Algorithm**
**All (0.5, 1, 1.5, 2, 2.5 h)**	Mean Imputation, Mode Imputation, Constant Removal, Standardization	Test-Budgeted Statistically Equivalent Signature (SES) algorithm with hyper-parameters: maxK = 2, alpha = 0.01 and budget = 3 * nvars	Support Vector Machines (SVM) of type C-SVC with Polynomial Kernel and hyper-parameters: cost = 0.001, gamma = 0.001, degree = 2
**2.5-h**	Mean Imputation, Mode Imputation, Constant Removal, Standardization	Test-Budgeted Statistically Equivalent Signature (SES) algorithm with hyper-parameters: maxK = 2, alpha = 0.01 and budget = 3 * nvars	Ridge Logistic Regression with penalty hyper-parameter lambda = 0.001
**2-h**	Mean Imputation, Mode Imputation, Constant Removal, Standardization	FullSelector	Support Vector Machines (SVM) of type C-SVC with Polynomial Kernel and hyper-parameters: cost = 0.001, gamma = 100.0, degree = 3
**1-h**	Mean Imputation, Mode Imputation, Constant Removal, Standardization	FullSelector	Ridge Logistic Regression with penalty hyper-parameter lambda = 0.001

Supplemental Table S2. Description of the best performing ML model pipeline using the 2 h gastric emptying value as a predictor for "flipping" from normal emptying at 2 h to abnormal (delayed) at 4 h.

Input Feature(s)	Model Pipeline		
	**Preprocessing**	**Feature Selection**	**Predictive Algorithm**
**2-h time point to "flip" from normal to delayed GE**	Mean Imputation, Mode Imputation, Constant Removal, Standardization	Test-Budgeted Statistically Equivalent Signature (SES) algorithm with hyper-parameters: maxK = 2, alpha = 0.1 and budget = 3 * nvars	Classification Random Forest training 1000 trees with Deviance splitting criterion, minimum leaf size = 5, splits = 1, alpha = 1, and variables to split = 0.577 sqrt (nvars)

Supplemental Table S3. Confusion matrices for the best performing models with the operating points set to maximize balanced accuracy, using all imaging time points as predictors (top) and using the 2.5 h only (bottom).

**Confusion Matrix Input Features: (0.5, 1.0, 1.5, 1.0, 2.5 h)**		**Predicted Class**	
		Class "0.0" = normal GE	Class "1.0" = delayed GE
**True Class**	Class "0.0" = normal GE	0.660 [0.611,0.710]	0.134 [0.098,0.175]
	Class "1.0" = delayed GE	0.029 [0.012,0.046]	0.178 [0.143,0.216]
**Confusion Matrix Input Feature: (2.5 h)**		**Predicted Class**	
		Class "0.0" = normal GE	Class "1.0" = delayed GE
**True Class**	Class "0.0" = normal GE	0.606 [0.360,0.742]	0.188 [0.064,0.435]
	Class "1.0" = delayed GE	0.028 [0.004,0.062]	0.179 [0.133,0.226]
